# *Drosophila* as a Model for Assessing the Function of RNA-Binding Proteins during Neurogenesis and Neurological Disease

**DOI:** 10.3390/jdb6030021

**Published:** 2018-08-18

**Authors:** Eugenia C. Olesnicky, Ethan G. Wright

**Affiliations:** Department of Biology, University of Colorado Colorado Springs, 1420 Austin Bluffs Parkway, Colorado Springs, CO 80918, USA; ewright3@uccs.edu

**Keywords:** *Drosophila*, RNA-binding proteins, neurons, neurodegeneration, amyotrophic lateral sclerosis, Fmr1, spinal muscular atrophy, TDP-43, FUS, *C9orf72*, shep, brat

## Abstract

An outstanding question in developmental neurobiology is how RNA processing events contribute to the regulation of neurogenesis. RNA processing events are increasingly recognized as playing fundamental roles in regulating multiple developmental events during neurogenesis, from the asymmetric divisions of neural stem cells, to the generation of complex and diverse neurite morphologies. Indeed, both asymmetric cell division and neurite morphogenesis are often achieved by mechanisms that generate asymmetric protein distributions, including post-transcriptional gene regulatory mechanisms such as the transport of translationally silent messenger RNAs (mRNAs) and local translation of mRNAs within neurites. Additionally, defects in RNA splicing have emerged as a common theme in many neurodegenerative disorders, highlighting the importance of RNA processing in maintaining neuronal circuitry. RNA-binding proteins (RBPs) play an integral role in splicing and post-transcriptional gene regulation, and mutations in RBPs have been linked with multiple neurological disorders including autism, dementia, amyotrophic lateral sclerosis (ALS), spinal muscular atrophy (SMA), Fragile X syndrome (FXS), and X-linked intellectual disability disorder. Despite their widespread nature and roles in neurological disease, the molecular mechanisms and networks of regulated target RNAs have been defined for only a small number of specific RBPs. This review aims to highlight recent studies in *Drosophila* that have advanced our knowledge of how RBP dysfunction contributes to neurological disease.

## 1. RBP Dysfunction Underlies Myriad Neurological Disorders

Neurological diseases, including neurodevelopmental and neurodegenerative disorders, represent one of the leading public health challenges of our time. In addition to the loss in quality of life associated with chronic neurological disorders, they also represent a significant economic burden due to the cost of medical treatment and the financial pressures incurred by patients and their caregivers. According to the National Institute of Neurological Disorders and Stroke, hundreds of different neurological disorders affect more than 50 million individuals within the United States. Yet for many of these diseases, a paucity of information exists about which basic molecular genetic mechanisms are affected in the disease. This significant gap in knowledge precludes effective treatments for patients. Defects in RNA-binding protein (RBP) functions, especially RBPs that are involved in alternative splicing, have, however, emerged as a common theme in a number of neurodegenerative disorders, including spinal muscular atrophy (SMA), amyotrophic lateral sclerosis (ALS), Frontotemporal lobar dementia (FTLD), and myotonic dystrophy (DM) [1,2,3,4,5,6,7]. This review, though not comprehensive, will highlight some of the recent knowledge gained from *Drosophila* regarding RBP function within the nervous system, and will discuss some of the unique tools available in *Drosophila* to study RBP dysfunction.

## 2. *Drosophila* Models of Neurological Disease

### 2.1. Drosophila Models of Amyotrophic Lateral Sclerosis (ALS)

Amyotrophic lateral sclerosis (ALS) is a common adult onset neurodegenerative disorder that results in the selective loss of motor neurons and consequently motor function. While the onset of disease is typically during midlife, most patients die within 3–5 years due to respiratory failure. The vast majority (~90%) of ALS cases are sporadic, while only a minority of cases are familial. Nonetheless, since both sporadic and familial ALS share many pathological features, much insight into disease progression and treatment can be gained from studying genetic models of ALS (reviewed in [8]). While mutations in genes encoding various types of proteins have been identified as causal for ALS, a number of the affected genes encode RBPs including TDP-43, FUS/TLS, and Heterogeneous nuclear ribonucleoprotein A1 and A2B1 (hnRNPA1 and hnRNPA2B1) (reviewed in [9]). *Drosophila* models have been established for many ALS-causing alleles and have even been recently generated to investigate risk factors associated with the development of sporadic ALS.

### 2.2. Modeling TAR DNA Binding Protein 43 (TDP-43) Mutations in Drosophila

With the advent of genome-wide association studies (GWAS), a large number of disease-associated alleles have been uncovered for various neurological disorders. Nonetheless, a major challenge for interpreting such data lies in determining whether such alleles are actually causative of the associated disease state. Moreover, understanding the molecular mechanism by which such mutations contribute to disease is paramount to developing effective therapeutics that are specifically tailored to a given patient’s genotype. *Drosophila* has proven to be invaluable in deciphering the contribution of various disease-associated alleles to disease phenotypes. This is especially true for diseases like ALS, which can be caused by mutations in a number of different loci, and for which many different disease-causing alleles have been identified for each locus.

**Figure 1:**
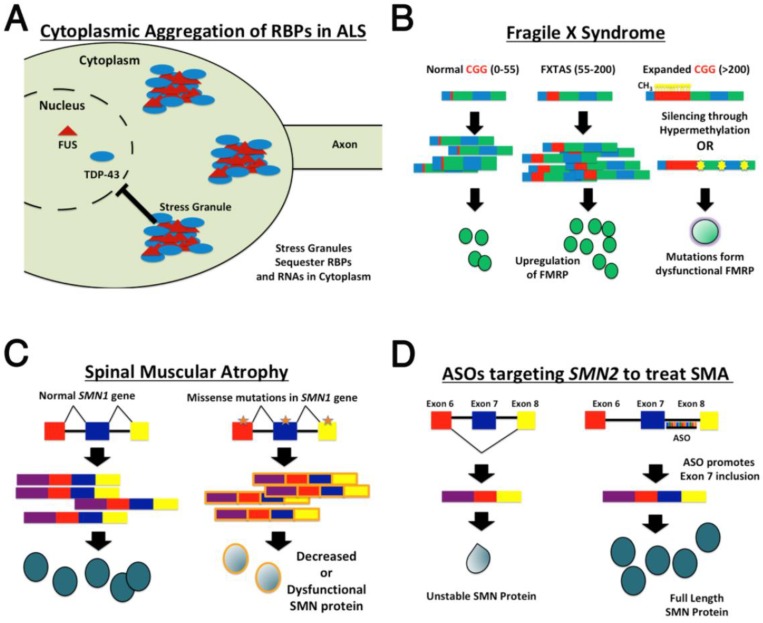
RBP dysfunction in neurological disease. (**A**) A subset of familial Amyotrophic lateral sclerosis (ALS) cases are caused by mutations in RBPs such as FUS and TDP-43. Aberrant forms of these proteins promote the formation of stress granules in the cytoplasm, sequestering RBPs and RNAs, and preventing the normal activities of FUS and TDP-43 in the nucleus; (**B**) Fragile X Syndrome (FXS) and FXTAS are caused by genetic lesions in the *FMR1* locus. FXTAS is caused by the expansion of CGG repeats in the 5′ end of the FMR1 such that individuals have between 55–200 repeats. This results in the upregulation of *FMR1.* Patients with FXS, on the other hand, have more than 200 CGG repeats in the 5′ region of *FMR1,* which results in silencing of the FMR1 locus through hypermethylation. Other cases of FXS are caused by point mutations or deletions in the *FMR1* locus that render FMRP nonfunctional; (**C**) Spinal muscular atrophy is caused by mutations in the *SMN1* locus, which decreases the amount of functional SMN protein; (**D**) However, new antisense oligonucleotide (ASO) technology has been developed to target *SMN2*, a paralog of *SMN1. SMN2* cannot normally compensate for loss of *SMN1*, because splicing of *SMN2* results in exclusion of exon 7, rendering the SMN protein unstable. ASOs have been developed to promote exon 7 inclusion during splicing of *SMN2*, thus increasing the amount of functional SMN protein.

For example, the human TAR DNA binding protein 43 (TDP-43) encoded by the *TARDBP* gene is an evolutionarily conserved gene that has been implicated in multiple neurodegenerative disorders including ALS, frontotemporal dementia, and Alzheimer disease [10]. A common theme in these diseases is that TDP-43 forms inclusions within the cytoplasm, with concomitant loss of TDP-43 from the nucleus (Figure 1). These observations have led to the formation of multiple hypotheses regarding the molecular nature of mutant TDP-43 forms, including gain of function toxicity of cytoplasmic inclusions, or, by contrast, loss of nuclear TDP-43 function. *Drosophila* has been used to investigate the molecular dysfunction of multiple ALS-associated *TARDBP* mutations, including the typical ALS-associated alleles p.G287S and p.A315T. Taking advantage of existing null alleles in the *TARDBP Drosophila* ortholog *TBPH,* researchers investigated the ability of these disease associated alleles to rescue various phenotypes associated with loss of *TBPH* function, including reduced lifespan and death of adult bursicon neurons. In both assays, each of the ALS-associated alleles failed to rescue the mutant phenotypes, providing evidence that the disease associated lesions act as partial loss-of-function alleles. Moreover, each of the two alleles was associated with a redistribution of TDP-43 from the nucleus to the cytoplasm, suggesting that the loss of function may be due, at least in part, to the depletion of TDP-43 from the nucleus [11].

While this study provides evidence for these particular patient mimetic alleles to have decreased function, other studies have provided evidence for gain of TDP-43 function as the underlying cause of neurodegenerative phenotypes [12]. Thus, the level and subcellular distribution of TDP-43 expression likely requires very strict regulation to prevent neuronal toxicity. Not surprisingly, TDP-43 expression is regulated through an autoregulatory feedback loop, where TDP-43 binds to a region of its own mRNA termed *TDBPR* and thereby regulates alternative isoform usage of *TDP-43* mRNA, as well as, its stability and subcellular localization [13,14]. A *Drosophila* model that investigates the regulation of TDP-43 expression through *TDBPR* was recently developed and it was used to identify six RBPs, namely Rsf1, B52, x16, SC35, Rbp1, and SF2 as regulators of TDP-43 expression [15]. Further investigation into how these factors regulate *TDP-43* isoform usage, and subcellular distribution may help provide new targets for therapeutics in multiple neurodegenerative diseases.

In some studies, work in *Drosophila* has generated new findings that have previously not been observed in ALS patients, but upon closer examination, show similar pathology in human patients. For example, work in *Drosophila* identified *futsch/MAP1B* as an in vivo TDP-43 RNA target in neurons [16]. Further work showed that TDP-43 regulates both the transport and translation of *futsch* mRNA, where TDP-43 specifically represses translation of *futsch* transcripts and TDP-43-induced toxicity results in the accumulation of Futsch within the soma of motoneurons. Interestingly, overexpression of Futsch was able to ameliorate TDP-43 toxicity phenotypes including reduced lifespan and neuromuscular junction (NMJ) morphology defects. Furthermore, the authors found that Futsch overexpression reduces the ability of TDP-43 to form aggregates. Finally, using postmortem tissue from the hippocampus and spinal cord, the authors confirmed that MAP1B also accumulates in motoneuron cell bodies in ALS patients but not in patients without neurological disease [16]. While more work is needed to understand whether accumulation of MAP1B in cell bodies causes neuronal toxicity, determining how TDP-43 dysfunction affects the expression of its target mRNAs is important for understanding why neurodegeneration occurs in response to aberrant TDP-43 function.

TDP-43 has also been shown to sequester and translationally repress the mRNA encoding Hsc70-4, a molecular chaperone involved in protein folding and stress response. This is consistent with many reports that TDP-43 is associated with stress granules and other types of ribonucleoprotein (RNP) complexes. Stress granules are a type of RNP that form in response to cellular stress, and serve to sequester and translationally silence RNAs. Importantly, co-overexpression of Hsc70-4 and TDP-43 in *Drosophila* ameliorates defects in locomotion and lifespan that are associated with TDP-43 overexpression. Moreover, Hsc70-4 overexpression reduces defects in synaptic vesicle endocytosis that are induced by TDP-43 overexpression [17], further highlighting the importance of understanding the consequences of TDP-43 dysfunction on its target RNAs.

**Figure 2:**
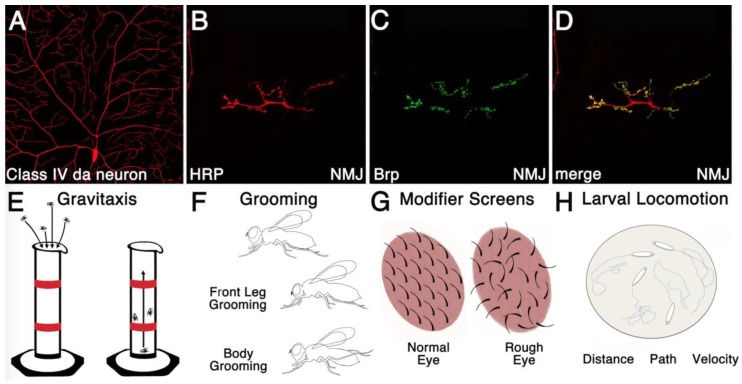
Phenotypic analyses to Assess RBP function within the nervous system. (**A**) Dendrite morphology can be easily assessed using live imaging of the highly complex dendritic trees of Class IV da neurons in larvae. In this image *ppkGal4* drives the expression of *UAStdtomato* to label the da neuron; (**B**) Axon morphology of the larval NMJ can be visualized through the dissection of larvae and subsequent immunofluorescent detection of horse radish peroxidase (HRP) (**B**) and the active zone marker Bruchpilot (Brp) (**C**) (merge shown in **D**); (**E**) Behavior can be investigated using gravitaxis assays, which measure the amount of time it takes for adult flies to walk up to a certain height in a cylinder (the start and stop heights are marked by a red line), after being tapped to the bottom; (**F**) Grooming assays are also used to assess stereotypic repetitive behavior. *Drosophila* grooming behavior involves cleaning the body and wings with their legs, as well as cleaning legs with mouthparts; (**G**) The morphology of the *Drosophila* eye provides a quick readout for genetic interactions during modifier screens. Researchers look for enhancement or suppression of a rough eye phenotype that arises through disordered development of the ommatidia, which make up the compound fly eye (**G**); (**H**) In addition to adult locomotor behavior, larval locomotion can be easily assessed by tracing the path, distance, and speed that a larva travels on a petri dish over a certain period of time.

In addition to its role in post-transcriptional gene regulation, TDP-43 has recently been implicated in the regulation of chromatin remodeling in *Drosophila*. One of the greatest strengths of the *Drosophila* system is the ability to perform high throughput genetic screens. The fly eye, in particular, has been utilized extensively to investigate genetic interactions and to perform modifier screens (Figure 2). The eye is used, in part, due to the ease of identifying aberrant eye morphology, and also due to the ability to probe otherwise lethal genetic interactions solely within the eye, without disrupting the development of other structures. Using a modifier screen in the fly eye, 31 genes with known functions in chromatin remodeling were uncovered as modifiers of TDP-43 induced toxicity. Further investigation showed that TDP-43 prevents recruitment of Chd1, a chromatin remodeling factor, to stress response genes such as *Hsp70*. This leaves cells that are unable to effectively respond to cellular stress, which might contribute to the development of neurological disease [18].

A link between TDP-43 and chromatin remodeling has also recently been uncovered in omega speckles of *Drosophila* motoneurons. Omega speckles are a distinct type of RNP complex that are found within the nucleus, which are characterized by containing the long architectural noncoding RNA heat shock omega (hsr**ω** arcRNA). TDP-43/TBPH binds hsr**ω** arcRNA in *Drosophila* neurons. Interestingly, the chromatin remodeler ISWI is important for maintenance of these omega speckles and modulates the interaction between TBPH and hsr**ω** arcRNA. Moreover, loss of ISWI function results in the redistribution of TDP-43 from the nucleus to the cytoplasm. Thus, chromatin remodeling may play a role in regulating the formation of specific TDP-43-containing RNP complexes and may influence their subcellular distribution [19], which is critical to normal TDP-43 function. Indeed, aberrant subcellular localization of various RBPs has emerged as a common theme in many neurodegenerative disorders.

### 2.3. Modeling Fused in Sarcoma (FUS) Mutations in Drosophila

Since mutations in the *TARDBP* locus account for a very small proportion of ALS cases, many groups have aimed to identify additional mutant loci that contribute to the development of ALS. Fused in Sarcoma (FUS) is an RNA-binding protein that functions in many aspects of RNA metabolism including splicing, RNA transport and translation. Mutations in the human *FUS* locus cause both frontotemporal dementia (FTD), and are associated with a small percentage of ALS cases. In both FTD and ALS, like TDP-43, FUS is often redistributed from the nucleus and forms aggregates within the cytoplasm that are thought to confer toxicity to neurons (Figure 1) [10]. A number of *FUS* animal models exist that have contributed immensely to our understanding of FUS-mediated neurodegeneration [8]. For example, knockdown of the FUS *Drosophila* ortholog *cabeza (caz)* results in locomotion defects in adults, as well as NMJ branch length defects in larvae [20]. Nonetheless, many of these models, although they recapitulate various aspects of ALS pathophysiology, do not result in the redistribution of FUS into cytoplasmic inclusions [21]. Recently, *Drosophila* sensory dendrite arborization (da) neurons were identified as a useful model for studying cytoplasmic FUS aggregates and their consequences, since overexpression of mutant forms of FUS or Caz results in the formation of cytoplasmic FUS/Caz aggregates, as well as neuronal phenotypes within these sensory neurons. Importantly, Machamer and colleagues found that ALS-associated mutant forms of FUS or Caz are actively transported within dendrites and axons, while the wild type FUS and Caz are undetectable, or barely detectable, in neurites. Overexpression of Fus or Caz forms results in progressive loss of dendrite and axon projections. Moreover, the ALS-causing forms of FUS/Caz disrupted transport of synaptic machinery in axons and resulted in neuronal hyperexcitability, which has previously been reported among ALS patients [21,22]. Thus, da neurons may serve as a particularly useful model for studying FUS cytoplasmic aggregation and its consequences on neuronal pathophysiology.

### 2.4. Modeling C9orf72 Aberrations in Drosophila

Supernumerary GGGGCC repeats in the first intron of the *C9orf72* locus represent the most common genetic cause of ALS, representing 40% of ALS familial cases [10]. Recently, the human RBP Znf106 was shown to bind to GGGGCC repeat-containing RNA [23]. This is of particular interest, since *Znf106* is located within a region of human chromosome 15 that has a strong linkage with a rare form of familial ALS [24]. Moreover, loss of function of the murine *Znf106* ortholog, *Zfp106,* results in neurodegeneration in adult mice [25]. Using a combination of experiments in mouse and *Drosophila*, Zfp106 was shown to play a conserved role in suppressing GGGGCC repeat-induced neurotoxicity. In *Drosophila,* overexpression of GGGGCC repeat-containing RNA within motor neurons using the Gal4/ *upstream activation sequence* (UAS) system results in geotaxis behavior deficits and a reduced number of active zones within the larval NMJ. However, co-expression of Zfp106 with GGGGCC repeat-containing RNAs ameliorates these phenotypes, suggesting that Zfp106 protects neurons from hexanucleotide repeat toxicity and may represent a potential therapeutic target for ALS [23].

A modifier screen in the fly eye was recently used to identify modifiers of toxicity resulting from dipeptide repeat proteins (DPRs) that are generated from transcripts arising from hexanucleotide repeat expansions within the *C9orf72* locus. Overexpression of DPRs results in neurodegeneration of the *Drosophila* eye confirming that DPRs are indeed toxic in the *Drosophila* adult [26]. Interestingly, overexpression of repeat containing RNAs that do not produce DPRs does not result in neurodegeneration or toxicity, although they are capable of forming cytoplasmic and nuclear foci that sequester RBPs [27]. This suggests that the repeat-containing RNA produced from the *C9orf72* locus is not toxic to neurons, but rather that DPRs mediate neuronal toxicity [27,28]. This is of particular note, since some studies in mouse models of *C9orf72* hexanucleotide expansions recapitulate cellular pathological ALS phenotypes, but do not result in neurodegeneration [29,30]. Notably, in these studies, DPR expression was lower than in other *C9orf72* hexanucleotide expansion models, suggesting that high levels of DPRs are required to produce neurodegenerative phenotypes. Since modulating gene expression levels is easily accomplished in *Drosophila,* the fly may be a particularly useful model for determining whether DPR concentration must pass a particular threshold to result in neurodegeneration.

Nonetheless, many groups have established that DPR toxicity phenotypes are common in many model organisms. Boeynaems and colleagues thus searched for genes that could enhance or suppress the DPR toxicity phenotypes when knocked down in the fly eye. The authors focused their efforts on 55 DPR modifier candidates that had previously been identified in a yeast genetic screen, and found that 19 of these genes were modifiers of DPR toxicity. A large number of these genes encode factors involved in regulation of transport across the nuclear membrane, as well as nuclear pore components. Additionally, knock down of a number of arginine methyltransferases enhanced DPR toxicity within the eye. Interestingly, the authors also found methylated protein aggregates within the cells of the dentate gyrus from a *C9orf72* frontotemporal dementia patient, suggesting that aberrant methylation may underlie disease pathology in ALS patients [26]. Future studies are needed to determine whether DPRs interfere with transport across the nuclear membrane, how DPRs globally modulate protein methylation, and whether DPRs directly affect arginine methyltransferase function.

While some groups have determined that GGGGCC-repeat-containing RNA is not responsible for neuronal toxicity, others have found that overexpressing such RNAs results in the formation of cytoplasmic granules that harbor the repeat containing RNA. Moreover, these granules are dynamic and they localize to neurites. Overexpression of these GGGGCC-repeat-containing RNAs in *Drosophila* sensory neurons results in degeneration of dendritic branches in late-stage wandering larvae. Interestingly, proteins that regulate mRNA localization, such as FMRP and Orb2, are modulators of this repeat toxicity. However, it is unclear whether the RNA itself or DPRs produced from these repeat-bearing RNAs are the cause of the neurodegeneration in sensory neurons [31].

Other studies have used strategies in the fly eye and wing for identifying modifiers of multiple ALS-linked mutations. Markmiller and colleagues sought to shed light on how stress granules contribute to neurodegeneration. Upon identifying conserved stress granule components, the research team utilized the fly eye and wing to determine whether the identified RBPs can modify Fus, TDP-43 and *C9orf72*-mediated toxicity. Using this strategy, the authors identified CBX3, CSDE1, RBMS1/2, UBAP2(L), and YEATS2 as novel stress granule components that can modify neurodegenerative phenotypes [32]. Thus, *Drosophila* is an effective model for studying multiple different types of genetic lesions that are known to cause ALS.

### 2.5. Modeling Sporadic ALS in Drosophila

While a small proportion of ALS cases are inherited, the vast majority (~90–92%) of ALS cases are sporadic [10]. Interestingly, inherited and sporadic ALS cases have strikingly similar cellular and physiological phenotypes, suggesting that they may share a molecular etiology. One well-established risk factor for developing ALS is repetitive head trauma. Recent studies have utilized *Drosophila* to begin to elucidate how repetitive head trauma contributes to the development of neurodegenerative disease. Anderson and colleagues established an experimental paradigm for inducing head trauma at both adult and larval stages, and examined the ability of such traumatic hits to induce stress granule formation [33]. Using green fluorescent protein (GFP)-tagged Rasputin (Rin) as a marker for stress granules, the authors found that repetitive head trauma induced stress granule formation. Moreover, the number and size of stress granules that formed increased proportionally with the number of traumatic hits. Importantly, TBPH/TDP-43 was associated with the stress granules that formed after traumatic injury. Additionally, subjecting FUS or *C9orf72* fly models of ALS to traumatic hits resulted in high mortality rates, thus showing that traumatic brain injury exacerbates phenotypes in genetic ALS models. Moreover, the authors found that pharmacological induction of autophagy was able to promote clearance of stress granules and increase survival among flies subjected to traumatic injury, providing a possible preventative measure or therapeutic option for patients with traumatic brain injury [33]. Taken together, these studies show the versatile tools that are available to probe the molecular etiology of ALS. Moreover, the studies show that *Drosophila* is not only an effective model for understanding how diverse genetic lesions lead to the development of neurodegeneration, but it can also be utilized to understand how head trauma may underlie sporadic ALS cases.

## 3. Modeling Fragile X Syndrome in *Drosophila*

### 3.1. Genetic Models of FXS

Fragile X syndrome (FXS) is the most common form of heritable intellectual impairment and the leading genetic cause of autism. FXS patients have myriad behavioral symptoms including cognitive impairment, altered circadian rhythm and sleep, abnormal social behavior, and repetitive behaviors. FXS patients also exhibit aberrant neurite morphology, and suffer from symptoms affecting nonneural tissues. FXS is most typically caused by a CGG repeat expansion within the 5′untranslated region (UTR) of the *Fragile X mental retartdation 1* (*FMR1)* locus, which results in epigenetic silencing of the *FMR1* gene (reviewed in [34]). FMRP functions predominantly as a translational regulator that binds over 800 target RNAs that are enriched in gene ontology terms related to neural tissue and synaptic transmission. Indeed, some of the RNA targets include autism candidate genes, consistent with FXS patients exhibiting autistic behaviors [35]. For example, FMRP translationally represses *Centg1* mRNA, which encodes PIKE, an enhancer of phosphoinositide-3 kinase signaling. Aberrant PI3K signaling is associated with various neuronal disorders including schizophrenia and autism. To determine whether elevated PIKE expression in *Fmr1* mutant animals underlies defects in neuronal development and behavior, Gross and colleagues examined whether reducing the dosage of *CenG1A* by half would rescue various neuronal phenotypes in *Fmr1* mutant flies. Indeed, reduction of *CenG1A* dosage rescued the fusion of axonal projections of the mushroom body associated with loss of *Fmr1* function, as well as short-term memory defects, as assessed through courtship suppression assays. Thus, enhanced translation of *CenG1A* encoding mRNA underlies at least some of the defects associated with loss of FMRP function [36].

Although most genetic tests for FXS only screen for repeat expansions or other larger aberrations within the locus by using Southern blot analysis, other loss-of-function *FMR1* mutations can also lead to the development of FXS (Figure 1). For example, a novel FXS-causing *FMR1* mutant allele was recently uncovered in a patient exhibiting FXS-like symptoms. The lesion in the *FMR1* locus stems from a frameshift mutation that causes a truncated protein with a novel nuclear localization sequence to be formed at its C terminus. Using the Gal4/UAS binary system, the authors overexpressed *Fmr1* or a patient mimetic *FMR1* construct in the *Drosophila* nervous system to begin to elucidate the molecular nature of the mutant allele. This strategy allowed the authors to determine that the allele has neomorphic properties that give rise to novel phenotypes, including axon guidance defects. Moreover, the patient mimetic protein is targeted to the nucleolus. Since FMRP is predominantly found within the cytoplasm, this change in its subcellular distribution may underlie some of the defects associated with this allele [37]. Similarly, a different patient-derived allele where a missense mutation leads to a partial loss of FMRP function was investigated using the fruit fly. Interestingly, this particular allele specifically disrupts FMRP function presynaptically, but does not compromise FMRP’s role in translational regulation. Thus, this allele provides an opportunity to understand FMRP’s role, specifically within presynaptic cells, as well as its molecular functions outside of translation [38].

### 3.2. Assessing Drug Efficacy in Treating Specific FXS Phenotypes

In addition to *Drosophila* serving as a tractable model for neuronal morphology and biochemical dissection of protein function, researchers have developed a large number of behavioral assays to probe the genetic regulation of behavior, enhancing the utility of *Drosophila* in investigating neurological disease (Figure 2). For example, a significant proportion of FXS patients exhibit autistic-like behaviors, which include stereotypic and repetitive behaviors. *Drosophila* may provide an excellent model for studying such FXS-associated repetitive behaviors and their potential therapeutics, since *Fmr1* mutant adult flies groom excessively compared to control animals (Figure 2) [39]. Moreover, the phenotype becomes progressively worse with age. Previous studies have shown that enhanced signaling through the metabotropic glutamate receptor (mGLuR) underlies some, but not all FXS associated phenotypes. Interestingly, mGLUR antagonists are unable to rescue the excessive grooming defects associated with loss of *fmr1* function in flies. However, antagonists of monoamine signaling are able to rescue excessive grooming in *Fmr1* mutants adults. Moreover, the expression of the *Drosophila* vesicular monoamine transporter, which loads monoamines into synaptic vesicles, is upregulated at both the mRNA and protein levels in *Fmr1* mutant animals. Taken together, these results suggest that monoamine signaling pathway modulation may provide a therapeutic avenue for treating repetitive behaviors [39]. This is of particular interest, since clinical trials using mGLuR5-antagonists were shown to be ineffective in treating FXS patients [40].

Additional studies show promise in targeting multiple different signaling pathways to treat FXS. For example, inhibition of signaling downstream of the BMP pathway, using various LIMK1 inhibitors, is able to rescue hyperactivity, aberrant locomotion, and abnormal NMJ morphology seen in *Drosophila* FXS models [41]. Similarly, the drug Acamprosate was recently investigated in a *Drosophila* FXS model for its efficacy as a therapeutic for molecular and behavioral defects. Acomprosate is thought to function as an inhibitor of glutamatergic signaling. Indeed, treatment of *Fmr1* mutant larvae with Acomprosate partially ameliorates defects in locomotor behavior, and rescues axonal overbranching defects within the NMJ. Interestingly, low and high doses of Acomprosate have varying effects on different phenotypes, suggesting that Acomprosate may have distinct, dose-dependent mechanisms of action. Thus, future studies in *Drosophila* may provide a greater understanding of the Acomprosate mechanism of action and its efficacy in treating FXS [42].

Recent studies have also postulated that aberrant GABAergic signaling might underlie some of the defects associated with loss of FMRP. *Drosophila Fmr1* mutant adults were shown to have a reduction in both attraction and aversion to specific odors. This results from dampening of odor coding and therefore a diminished capacity for odor discrimination. Although glomerular morphology and size is unaffected in *Fmr1* mutant animals, lateral inhibition between glomeruli is compromised in mutant animals. Downregulation of GABA signaling produces similar olfactory behavior defects, but a definitive link between GABA signaling and FMRP has not been established in olfactory behavior [43].

### 3.3. FMRP Function in Neuronal Remodeling

One of the greatest advantages of *Drosophila* is the diverse suite of tools available for manipulating gene expression, in both a spatial and temporal manner. This, combined with the ability to optogenetically manipulate neuronal activity, has allowed researchers to investigate the requirements of specific genes in an incredibly precise and sophisticated manner. For example, FMRP has been shown to regulate activity-dependent refinement of dendrites during critical period development. Using multiple Flylight lines, which allow researchers to visualize and genetically manipulate very specific subsets of neurons and even single neurons within the brain, *Fmr1* was shown to be required specifically during a critical period (on day one post-occlusion) to regulate refinement of dendrites in mushroom body neurons. Loss of *Fmr1* function leads to overelaborated dendritic arbors in mushroom body neurons. Using a temperature sensitive Gal80, the authors were able to specifically manipulate *Fmr1* only during the critical period to show that FMRP is required in a short window of time to facilitate neurite remodeling. Moreover, while optogenetic stimulation of these neurons during the critical period phenocopies the overelaboration seen in *Fmr1* mutant animals, optogenetic stimulation does not cause additional elaboration in *Fmr1* mutants, showing that this refinement process is activity dependent and it relies on FMRP to regulate neurite remodeling [44]. FMRP regulation of critical period remodeling occurs through the regulation of calcium signaling [45]. Interestingly, *Fmr1* mutant animals also show defects in immune cell-mediated remodeling of neurons in the mushroom body, as well as axonal clearance after injury [46]. This suggests that defects within glia, the immune cells of the nervous system, may also underlie defects in neurite refinement.

### 3.4. Modeling Fragile X-Associated Tremor Ataxia Syndrome FXTAS

In contrast to Fragile X Syndrome, Fragile X-associated tremor ataxia syndrome (FXTAS) is a heritable neurodegenerative disorder where patients over the age of 50 develop tremors, ataxia, and dementia. FXTAS results from intermediate CGG repeat expansions (50–200 repeats) in the *FMR1* locus, which causes the upregulation of *FMR1*, as well as the accumulation of CGG-repeat containing mRNAs. Multiple groups have postulated that the disorder arises primarily through an RNA toxicity mechanism, while others have hypothesized that, instead, repeat-associated non-AUG (RAN)-initiated translation produces toxic FMRP with polyglycine residues (FMRPpolyG) that mediate toxicity. To test which of these two hypotheses are correct, Yoon Oh and colleagues made use of *Drosophila* lines that express the CGG repeat mRNA but failed to make FMRPpolyG, due to a stop codon that precedes the CGG repeats. In addition, the group utilized a line where FMRPpolyG production is enhanced by the addition of an AUG start codon just upstream of the CGG repeat sequence. Expressing the CGG repeat RNA construct that does not produce FMRPpolyG in the eye results in a very mild rough eye phenotype. By contrast, expressing the FMRPpolyG-producing construct produces a more severe eye phenotype. Moreover, concomitant disruption of the ubiquitin proteasome system dramatically enhances the FMRPpolyG eye phenotype, and results in necrosis within the eye. These results suggest that RAN translation of FMRPpolyG mediates toxicity in neurons, and plays a stronger role in disease pathology than the CGG repeat-containing mRNAs alone [47]. This is consistent with work in *C9orf72* models of ALS, which show that DPRs generated from hexanucleotide repeat-containing RNA cause neuronal toxicity. Thus, the sophisticated tools available in *Drosophila* to precisely manipulate gene expression provide an excellent platform for studying various neurological disorders related to FMRP dysfunction.

## 4. Spinal Muscular Atrophy

Spinal muscular atrophy (SMA) is the leading heritable cause of infant mortality and is one of the most common autosomal recessive genetic disorders. It is defined by the degeneration of motor neurons in the spinal cord, and progressive loss of motor function. Combined with the loss of motor neurons, SMA patients also suffer from the progressive loss of muscle mass [48]. SMA patients are classified into four categories, which reflect the age of disease onset and severity of motor dysfunction. For example, SMA Type I is the most severe form, with a typical onset during infancy and results in death within the first two years of life. By contrast, SMA IV first develops during the second or third decade of life, and symptoms mainly include mild muscle weakness. SMA largely results from homozygous lesions in the *Survival Motor Neuron 1 (SMN1)* locus. *SMN1* encodes the RBP SMN, which functions in the biogenesis of small nuclear ribonucleoproteins (snRNPs), and has been implicated in pre-mRNA splicing and axon guidance [49,50]. *SMN1* lesions typically result in a reduced level of SMN protein (Figure 1). Interestingly, the severity of SMA is correlated with the level of SMN expression, where modest reductions in SMN protein levels typically result in less severe disease pathology (reviewed in [49]).

### 4.1. Drosophila Models of Spinal Muscular Atrophy

In *Drosophila,* animals homozygous for a null allele of *Smn* have decreased viability, aberrant locomotion, decreased muscle size, aberrant motor neuron transmission, and reduced small nuclear RNA (snRNA) levels [50,51]. Having a *Drosophila* model that recapitulates many of the symptoms of SMA allows researchers to investigate the molecular nature of diverse patient derived *SMN1* alleles, many of which provide a wealth of information about SMN’s molecular functions. For example, in an attempt to characterize the patient derived allele, *SMN^T274I^*, Praveen and colleagues found that although this patient mimetic allele is capable of rescuing snRNA levels, it is unable to rescue pupal lethality, suggesting that SMN’s role in snRNP biogenesis is dispensable for the development of SMA. Instead, SMN functions outside of snRNP biogenesis may underlie neurological disease phenotypes [50]. Still, others have shown that overexpression of WDR79, which regulates snRNAs within Cajal bodies, can rescue neural phenotypes associated with loss of Smn function [52]. Nonetheless, it is not clear if snRNA related functions of WDR79 are relevant to this interaction.

Since over 25 different *SMN1* point mutations have been identified among SMA patients, additional studies have taken a rescue approach to study multiple patient mimetic alleles to determine their severity and the molecular nature of each disease-associated allele. One study examined 12 different patient mimetic alleles that were found to have different effects on SMN function. For example, some alleles affect the stability of SMN, while others affect SMN self-oligomerization or compromise interactions with known binding partners, such as members of the Gemin family. Moreover, while some *SMN* patient mimetic alleles are inherited in a recessive manner, others affect SMN binding with itself or other RBPs during RNP assembly, and thus function in a dominant negative manner. This is of particular importance, as these dominant negative alleles may be refractory to certain SMA therapeutics. Investigation of these patient mimetic alleles has also provided insight into the onset of symptoms and the life expectancy in patients bearing such alleles. Continued work to understand the molecular nature of newly discovered patient mimetic alleles will have profound implications on generating genotype-specific therapeutic approaches to treat SMA [53].

Since SMN is ubiquitously expressed, efforts have been made to determine whether SMN function is required within the nervous system, muscle, or multiple cell types, to develop and maintain NMJ morphology and function. *Drosophila* is particularly useful for investigating tissue specific gene functions, as there are a wealth of tools, including extensive collections of Gal4 driver stocks, which allow researchers to manipulate gene expression in a cell type-specific manner. Using tissue specific expression of *SMN* rescue constructs, Imlach and colleagues found that SMN function within muscles is dispensable for regulating muscle size, larval locomotion, motor rhythm, and motoneuron transmission. Moreover, while pan-neuronal expression of an *SMN* rescue construct is sufficient to rescue these phenotypes, expressing SMN in motor neurons alone is unable to rescue these defects. Instead, SMN function is required in proprioceptive and central cholinergic neurons for NMJ associated phenotypes [51]. Similarly, RNA interference (RNAi)-mediated knockdown of *Smn* throughout the nervous system, but not within muscle, results in adult locomotor phenotypes. In contrast to how tissue-specific manipulations of *Smn* function affect larval locomotion, knockdown of *Smn* specifically within motor neurons, does result in aberrant locomotion in adults. This suggests that Smn’s requirements within specific cell types may change throughout development [54]. However, the tissue specific requirements of Smn remain contentious, since other groups have shown that manipulating Smn levels using stronger muscle drivers, namely *how*-*Gal4*, causes aberrant NMJ morphology and lethality [55]. Moreover, *Smn* and *Gemin3* genetically interact within muscle to affect viability and adult locomotion [56]. Taken together, these studies highlight the need to use multiple different tools to manipulate gene expression to reliably determine the tissue specificity of Smn function.

One advantage of using RNAi to knockdown Smn expression is that it results in only a partial knockdown, allowing researchers to examine adult phenotypes, whereas *Smn* null alleles result in lethality and preclude the ability to investigate adult NMJ and locomotor phenotypes. Investigating Smn function in adults is of particular interest, since some evidence has suggested that Smn and FUS may function together. Nonetheless, a genetic interaction between *Smn* and *FUS* was not detected in one study using the fly eye. Thus, future studies are required to determine if FUS may instead act downstream of Smn [57].

RNAi-mediated knockdown of *Smn* has also been useful in creating sensitized background for identifying modifiers of Smn function. Using this approach, an estimated 340 *Drosophila* genes were identified as candidate modifiers. Importantly, many of these modifiers are conserved in humans and correspond to 322 human genes. Further examination of 20 of these conserved candidates revealed that, in addition to modifying pupal lethality phenotypes, 11 genes are also modifiers of the *Smn* NMJ phenotype, and seven affect Smn expression [58]. Thus, such modifier screens, coupled with secondary screening of evolutionarily conserved candidates, provide a promising pool of potential human SMN modifiers that may contribute to disease progression and may represent therapeutic targets.

### 4.2. Determining the Efficacy of SMA Therapeutics Using Model Organisms

In 2016, the first antisense oligonucleotide (ASO) treatment for SMA, nusinersen, became available to patients [59]. This ASO treatment very cleverly takes advantage of the partially functional human *SMN1* paralog, *SMN2*. *Smn2* is unable to compensate for *Smn1* mutations due to a single nucleotide change in the *Smn2* locus that causes a different SMN splice form that lacks exon 7 to be favored. This particular isoform that lacks exon 7 is degraded, while the full-length SMN isoform is expressed at extremely low levels (Figure 1) [60]. Nusinersen specifically binds to the *Smn2* transcript and promotes the inclusion of exon 7, thus increasing SMN protein levels. The results of the nusinersen clinical trials have been impressive, to say the least. Nonetheless, it is not clear if the ASO treatment will be effective for patients harboring alleles that function in a dominant negative manner. Moreover, while nusinersen has been incredibly successful in treating SMA patients, its cost is prohibitive for many patients who would pay $750,000 for the first year of treatment and $375,000 each subsequent year [59,61,62]. Thus, while nusinersen represents an incredible step in the treatment of SMA and more widely in the use of ASOs as therapeutics, additional studies aimed at identifying other effective treatments are critical. Moreover, combining nusinersen with additional therapeutics may increase the efficacy of treatment and quality of life in certain patients.

*Drosophila* provides opportunities to test pharmacological treatment of SMA-related neurological phenotypes. For example, SMA dysfunction results in aberrant ubiquitination of various proteins, including β-catenin. This leads to the accumulation of β-catenin in mouse models. However, treatment with quercetin, an inhibitor of β-catenin signaling, is sufficient to rescue NMJ morphology defects in mouse, zebrafish, and flies [63]. The ability of quercitin to ameliorate NMJ defects in three different model systems, including both invertebrate and vertebrate SMA models, provides great promise in its therapeutic potential to treat SMA in humans. Thus, *Drosophila* SMA models have the potential to be useful for relatively quick and simple pharmacological screens that are designed to rescue *Smn^-/-^* lethality and NMJ phenotypes. Promising candidates can then be screened more rigorously in vertebrate organisms to identify additional bona fide human SMA therapeutics.

## 5. Additional Roles for RBPs throughout *Drosophila* Neurogenesis

In addition to work on RBPs that have been specifically implicated in neurological disease, many studies have uncovered important roles for RBPs and RNA processing events throughout various stages of neurogenesis [64,65,66,67,68,69,70]. Research on RBP function during neurogenesis is integral to understanding the global roles of RBPs within the nervous system. Moreover, such studies are able to shed light on how RBP dysfunction underlies various neurological diseases, including neurodegeneration and brain cancers. For example, it has been well established that asymmetric localization of mRNAs and proteins is critical to maintaining a normal balance between self-renewal and differentiation in *Drosophila* neural stem cells, termed neuroblasts [65,71]. During the process of neuroblast division, the cell fate determinant Prospero must be preferentially segregated into the ganglion mother cell (GMC), which will differentiate into neurons or glia. Prospero must, however, be excluded from the neuroblast, such that it can continue to self-renew [72,73]. This asymmetric segregation of Prospero is achieved, in part, by the RBP Staufen binding to *prospero* mRNA. Subsequently, this complex is asymmetrically localized by the scaffolding protein Miranda, which tethers Staufen, *prospero* mRNA, and Prospero protein to the basal cortex of the dividing neuroblast [74,75,76].

Other RBPs have also been implicated in regulating neuroblast differentiation into distinct neural subtypes. The RBPs IGF-II mRNA-binding protein (Imp) and Syncrip (Syp) form opposing temporal gradients, where Imp promotes the formation of early neural cell fates, and Syp favors late neural cell fates within the Mushroom Body of the central nervous system [77,78]. Concomitantly, Imp and Syp regulate the ‘decommissioning’ of neural stem cells, which facilitates cell cycle exit of individual neuroblasts, and consequently the termination of those neuronal lineages [79]. Additionally, the splicing factor Barricade (Barc) has also been implicated in regulating neuronal proliferation and differentiation. Loss of Barc function results in supernumerary neural precursor cells, at the expense of terminally differentiated neurons, which causes neuroanatomical defects in the adult brain [80].

Much work has also been dedicated to determining how the dysfunction of the RBP Brain tumor (Brat) results in overproliferation of neuroblasts and consequently overgrowth of the *Drosophila* brain [81]. During neuroblast development, Brat functions to regulate neuroblast proliferation, in part, by suppressing Notch signaling [82] and through the post-transcriptional repression of *deadpan* and *zelda* mRNAs [83]. Moreover, the dysregulation of the RBPs Imp and Lin-28 contribute to tumor formation in *brat^−/−^* neural tissue [84]. Loss of *brat* also results in dysregulation of the long noncoding RNA *cherub. cherub* is typically asymmetrically segregated into intermediate neural precursor cells. However, upon *brat* depletion, *cherub* accumulates in tumorigenic neuroblasts and results in aberrant subcellular distribution of the RBPs Staufen and Syp, leading to defects in the differentiation program [85]. It has subsequently been found that mutations in *TRIM3,* the human ortholog of *brat*, are associated with 25% of glioblastoma cases, highlighting the utility of *Drosophila* in studying the regulation of neural stem cell division and differentiation [86].

In addition to the extensive work done on RBP regulation of neural stem cell differentiation, RBPs have also been implicated in regulating neurite morphogenesis. Understanding how RBPs regulate neurite morphogenesis, and particularly maintenance of neurite morphology during development can help shed light onto why RBP dysfunction can lead to the degeneration of neural processes in neurological diseases. For example, it is well established that the RBPs Nanos (Nos) and Pumilio (Pum) function in the development and maintenance of dendritic branching in the morphologically complex Class IV da neurons [87,88]. Nos and Pum regulate dendrite morphology and dendritic dynamics by post-transcriptionally repressing the mRNA encoding the proapoptotic factor Hid, which maintains a balance between neurite outgrowth and retraction [89]. Moreover, *nanos* mRNA is localized to dendrites by the RBPs Rumpelstiltskin and Oskar, and this localization is required for normal dendritic elaboration [90,91].

**Figure 3:**
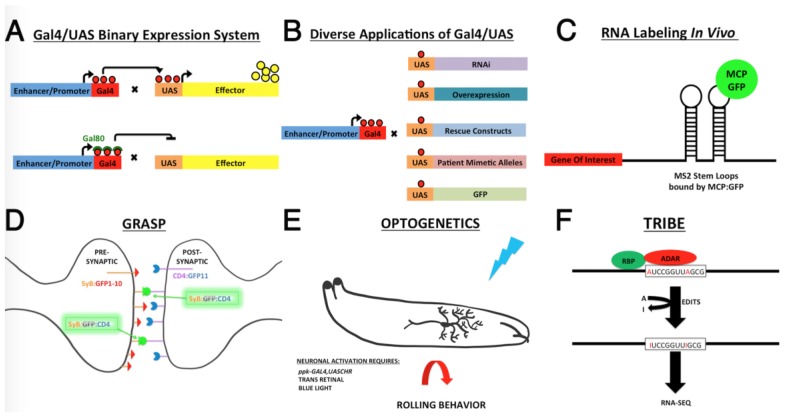
Tools available in *Drosophila* to facilitate the dissection of RBP function within the nervous system. (**A**) The Gal4/UAS binary expression system can be combined with temperature-sensitive Gal80 to control gene expression in a spatial and temporal manner. Under permissive temperatures (25 degrees Celsius) Gal80 prevents Gal4 activation of UAS. However, at restrictive temperatures (29 degrees Celsius), Gal80 is inactivated, allowing Gal4 to activate UAS constructs; (**B**) UAS-effector lines can mark cells with Grenn Fluorescent Protein (GFP), can cause overexpression of genes, RNA interference-mediated knockdown of gene expression, can assess the molecular nature of patient specific alleles, or express rescue constructs; (**C**) mRNA dynamics can be visualized within neurons of live animals using the MS2/MCP labeling system. This requires the insertion of MS2 stem loops into a gene of interest. The MS2-tagged RNA and the MS2 Coat Protein (MCP) fused to GFP are then expressed using the Gal4/UAS system. Since MCP:GFP specifically binds to MS2 stem loops, and the tagged mRNA becomes illuminated; (**D**) Synaptic partners within a neural circuit can be visualized using GRASP, which requires adjacent cells to make close contact to reconstitute GFP fluorescence from two different fragments of GFP that are tethered to the synaptic membrane (Synaptobrevin:GFP1-10 or SyB:GFP1-10 and CD4GFP). Moreover, the presence of GFP fragments within the synaptic cleft is dependent upon neuronal activity; (**E**) Functional neurons within a circuit can be assessed using optogenetic tools to activate a neuron and assess a behavioral readout, such as the ability of a larva to roll in response to the optogenetic activation of a Class IV da neuron. In this case, *ppkGal4* can be used to drive expression of *UASChannel rhodopsin* in da neurons. Rearing animals on trans-retinal and exposing them to blue light would result in activation of these neurons and cause the larva to roll; (**F**) “targets of RNA-binding proteins identified by editing” (TRIBE) allows researchers to identify target RNAs of a specific RBP in a tissue specific manner by leaving A to I edits within the RNA, which can later be identified through sequencing and bioinformatics. For such experiments, an RBP is fused to the editing domain of the ADAR protein, allowing the RBP to bind to its endogenous RNA targets but leave A to I edits near the binding site though the catalytic ADAR domain.

A screen for RBPs and translation factors that are specifically required for dendrite development within Class IV da neurons uncovered 88 candidate genes that regulate dendrite morphology in Class IV da neurons [92]. Subsequent studies have confirmed that a number of these candidate genes, including *brat, shep, caper, 4EHP, oskar* and *rumpelstiltskin,* indeed function in da neuron morphogenesis, and that 12 of these RBPs play a conserved role in dendrite development in *C. elegans* [88,93,94,95]. The fact that almost all of these candidates are conserved across metazoa suggests that RNA regulatory mechanisms are critical to the development and function of the nervous system. This idea is also supported by the fact that a large number of RNAs are localized to neurites in many different species [96,97,98,99,100,101,102,103,104]. Importantly, the ability to visualize specific RNA transcripts in vivo, using the MS2/MCP in vivo fluorescent labeling system, makes *Drosophila* an ideal model for investigating the regulation and dynamics of mRNA localization within neurons [90,91,96]. Indeed the MS2/MCP labeling was recently combined with *EP* transposon insertion to perform a genome-wide screen to identify novel dendritically localized RNAs (Figure 3). This strategy identified 55 dendritically localized transcripts. Moreover, knockdown of many of the genes encoding these dendritically localized transcripts resulted in dendritic defects, underscoring the utility of this unbiased screening approach to identify new players in dendrite morphogenesis [96].

RNA localization also commonly occurs within axons, and RBPs, which direct the localization and translation of these localized RNAs, have been shown to regulate axon morphology and function. For example, the RBPs Nos, Pum, Cup, Orb, and Brat have been implicated in regulating different aspects of NMJ morphology and physiology in *Drosophila* larvae [88,105,106,107,108]. Interestingly, while Pum and Nos function coordinately during dendrite development in sensory neurons, they instead have opposing functions in NMJ morphogenesis.

The RBP Shep was recently shown to regulate axonal morphology during neuronal remodeling by regulating BMP signaling, as well as, the expression of Brat [86,109,110]. Not surprisingly, Brat has also been implicated in regulating axon maintenance within the central nervous system. In Mushroom Body axons, Brat translationally represses *src64B* mRNA. Src64B has previously been shown to promote axon retraction. Thus Brat stabilizes axonal morphology by preventing retraction pathways from being activated. Interestingly, Brat also functions in concert with Pum and Nos to direct dendrite development, yet its role in axonal maintenance is independent of Nos or Pum [88,111]. These findings suggest that RBPs may work coordinately in a cell-type specific manner, or that their interactions may even depend on their subcellular location within a single cell. Future work on the roles of additional RBPs in regulating neurite outgrowth and retraction will undoubtedly shed light on the extent to which RBPs are required to maintain neurite morphology, and how this might underlie neurodegeneration in RBP-associated neurological disease.

## 6. A Unique Toolkit for Investigating RBP Function within Neurons

The study of RBP function within the nervous system requires a sophisticated toolkit that allows for precise spatio-temporal genetic manipulations, a vast array of tools for the visualization of neuronal morphology, as well as a variety of behavioral assays to probe neurological function. *Drosophila* offers an incredible toolkit for performing genetic manipulations within the nervous system and investigating their consequences on neuronal function and physiology. Below, we will highlight just a few of the basic tools, as well as some emerging technologies, that make *Drosophila* an outstanding model for neurogenetics.

### 6.1. Precise Spatiotemporal Manipulation of RBP Function

Among *Drosophila*’s greatest strengths is the incredible number of transgenic lines that are available for manipulating gene expression in a spatial and temporal manner. The Gal4-UAS binary expression system was among the first technologies developed to control gene expression in a precise temporal and spatial manner [112]. This system comprises both a Gal4 ‘driver’ line and a UAS effector line that must be combined in order to manipulate gene expression. Thousands of Gal4 driver lines have been generated, in which the open reading frame for the yeast transcription factor Gal4 is placed downstream of a gene specific enhancer and promoter, which confers a particular expression pattern. Since each Gal4 driver line is controlled through a different enhancer, thousands of different expression patterns can be achieved. The UAS effector line, on the other hand, has a particular DNA sequence downstream of an *upstream activation sequence* (UAS) to which Gal4 binds to activate transcription (Figure 3). This sequence could be the open reading frame of a gene to allow overexpression of a gene, a hairpin sequence to knockdown gene expression, or simply the open reading frame for GFP, to be used as a cell marker. Once these two components are combined in the same fly through standard genetic crosses, the effector line will be activated in all cells that express Gal4 [112,113,114]. To add another layer of temporal control of gene expression, the Gal4/UAS system can be combined with a temperature-sensitive Gal80 construct. Gal80 binds to Gal4 and prevents it from activating the UAS effector line. However, by utilizing a temperature-sensitive Gal80, one can shift flies to the restrictive temperature (29 °C) to inactivate Gal80, and thereby allow Gal4 to activate UAS expression. Other variants of the Gal4 system, such as GeneSwitch or SplitGal4, have been developed to provide even more precise control of gene expression [114,115]. Another binary gene expression system, termed LexA-LexAop, was also developed, which when combined with the Gal4/UAS system, allows researchers to manipulate the expression of two different genes simultaneously [116]. In addition to such tools for manipulating gene expression, efforts have also been made to generate collections of fly lines that recapitulate the endogenous expression patterns for thousands of genes in the *Drosophila* genome. For example, *MiMIC* (Minos-mediated Integration cassette) lines utilize a GFP protein trap to provide information about a gene’s expression pattern, but also provide a platform for manipulating gene expression since the transposon can be modified after it has inserted into the genome [117]. With enormous collections of Gal4 and LexA drivers, UAS effector lines and *MiMIC* lines, gene manipulation has become remarkably simple for the *Drosophila* geneticist.

### 6.2. RNA Editing Tools to Identify Tissue Specific RBP Target RNAs

One of the major priorities in the field is to systematically identify RNA targets for the majority of RBPs expressed in the nervous system. The newly developed “targets of RNA-binding proteins identified by editing” (TRIBE) technique [118] enables researchers to identify in vivo target RNAs for an RBP of interest, in a tissue-specific manner (Figure 3). While multiple techniques that involve cross-linking followed by RNA immunoprecipitation (CLIP) have been generated in the past decade to identify direct RBP target RNAs, the low efficiency of crosslinking, especially in deeper cells, limits its use in embryos or brains [119]. To identify in vivo RNA targets using whole embryos or tissues, RNA immunoprecipitation (RIP) without crosslinking has generally been used instead. However, without the stringent washes that are performed after crosslinking, RIP often results in the identification of both direct and indirect target RNAs, and may also identify non-specific interactions [120]. The TRIBE technique, however, allows for the identification of target RNAs in a tissue-specific manner without the need for crosslinking or immunoprecipitation. In TRIBE, Gal4/UAS is utilized to provide tissue-specific expression of an RBP of interest fused to the catalytic domain of the RNA editing enzyme ADAR. ADAR edits A bases to I (read as G in a sequencing reaction), thus leaving permanent marks in bound RNAs that are specific to the RBP of interest. Since the ADAR RNA-binding domains are not included in this construct, RNA target specificity is determined by the RBP of interest. These marks can later be identified through sequencing and bioinformatics analysis of the resulting RNA libraries as compared to controls. Importantly, the TRIBE technique is successful in identifying direct in vivo RNA targets within cell types composed of as few as 150 cells per fly upon *fluorescence-activated cell sorting* (*FACS*) sorting [118]. One can easily imagine probing combinatorial regulation of a single mRNA target by multiple RBPs using multiple TRIBE constructs simultaneously. Moreover, UAS-TRIBE constructs can be activated in specific tissues to determine whether ubiquitously expressed RBPs regulate distinct RNA targets depending on tissue type. While TRIBE has only been used in a small number of studies to date, its potential applications are vast and may very well be transformative for *Drosophila* researchers.

### 6.3. Emerging Tools for Mapping Neural Circuitry

Although probing the function of RBPs in individual neurons is an important first step in understanding how RNA processing contributes to neuronal morphogenesis and maintenance, ultimately, investigation of how RBP dysfunction affects entire neural circuits will be necessary to draw a complete picture of how such perturbations affect brain function and organization at the organismal level. One major challenge in studying neural circuits is the ability to accurately map functional synapses. This is due to the incredibly complex morphology of neurons, elaborate wiring patterns, and the limitations of microscopy techniques. A recent technology developed in *C. elegans,* termed “GFP reconstitution across synaptic partners” (GRASP), provides a reliable and robust way to detect synaptic connections (Figure 3). The GRASP technique requires the expression of two complementary fragments of the GFP protein fused to a transmembrane domain in adjacent cells. If the two cells make close contact with one another, GFP fluorescence can be readily detected in live animals [121]. The GRASP technique has been further optimized and applied to *Drosophila*, where GFP fragments are specifically fused to synaptic proteins such as Synaptobrevin (termed syb:GRASP) [122,123]. Importantly, such changes to the GRASP technique only allow GFP fragments to be reconstituted upon vesicle fusion at the synaptic cleft, thereby making GFP fluorescence between synaptic partners activity-dependent. This is an important adjustment to GRASP technology, as neuronal processes can make many transient contacts that might normally be eliminated during development, but could be inappropriately stabilized by the reconstitution of GFP. Thus, syb:GRASP facilitates the visualization of active synapses in a live animal. Further development of multi-colored GRASP constructs, termed X-RASP and CRASP, has expanded the potential for simultaneously visualizing multiple synaptic connections [122,123].

The combination of GRASP and optogenetic neural activation provides a robust experimental paradigm for mapping functional neural circuits. In “optogenetics”, the light-activated cation channel, Channelrhodopsin (ChR), is expressed in a specific subset of neurons using the Gal4/UAS system (Figure 3). When animals reared on food containing trans-retinal are exposed to blue light, ChR-expressing cells become activated [124,125]. Optogenetics was recently used, in combination with GRASP, to determine the neural circuitry that regulates escape behavior in *Drosophila* larvae [126]. The authors were able to map contacts among Class IV da neurons, interneurons termed mCSIs and motor neurons using GRASP. Optogenetic activation of these subsets of neurons further confirmed that these neural subtypes are part of a neural circuit that induces a stereotypical rolling behavior used as an escape response by larvae [126]. Future studies that combine these tools will to help shed light on how neural circuits are affected during different stages of neurodegeneration.

## 7. Outstanding Questions and Conclusions

While it is well established that RBPs play a fundamental role in neurogenesis, surprisingly, only 2–6% of RBPs show tissue-specific expression in humans; fewer still are expressed specifically within neurons. This is despite the fact that the brain shows higher levels of splicing and other forms of RNA regulation than other tissues [2,127]. Moreover, dysfunction of ubiquitously expressed RBPs often results in tissue-specific pathologies, which often more severely affect the PNS as compared to other tissues [127]. Despite the fundamental importance of RNA processing in the development, function and maintenance of the nervous system, relatively few RBPs have been studied in detail, especially with regard to their neural functions. Therefore, a major goal of the field is to understand the neural-specific roles and neural specific target mRNAs of widely expressed RBPs. Moreover, in the case of splicing factors, it is critical to identify the specific isoforms of target mRNAs that are relevant to neurogenesis and neurodegeneration. It will be important to determine whether different isoforms have distinct functions within neurons, and whether a balance of isoforms is important for neural development. Moreover, a better understanding of which mRNA isoforms are important within the nervous system is critical, not only for determining why neural tissue is sensitive to splicing defects, but also for identifying potential therapeutic targets in neurological disease. A number of therapeutic strategies that take advantage of ASOs to correct splicing defects have recently been developed for treating SMA and DM. Importantly however, in both instances, the defective isoforms that contribute to the development of these specific diseases are already known [128]. Identifying other specific transcript isoforms that are necessary for development and neuronal maintenance is a critical step for the treatment of diseases resulting from RBP dysfunction, which potentially affects alternative isoform regulation of thousands of target RNAs.

In conclusion, multiple neurodegenerative disorders arise from the dysfunction of specific RBPs or RNA regulatory mechanisms, suggesting that RBPs play a fundamental role in nervous system function and development. Nonetheless, since many RBPs are widely expressed and their dysfunction can lead to lethality, it is not trivial to dissect their tissue-specific functions or target RNAs. However, due to the high level of conservation of RBPs across metazoa, the use of sophisticated genetic techniques in the highly tractable *Drosophila* model system provides a unique opportunity to study the roles of RBPs within the nervous system in vivo. In particular, the large number of publically available stocks, ease of generating transgenics and of performing tissue-specific molecular genetic manipulations in *Drosophila,* makes it an unparalleled system for the study of RBP functions within the nervous system.
